# Standardizing default electronic health record tools to improve safety for hospitalized patients with Parkinson’s disease

**DOI:** 10.3389/fnagi.2023.1278322

**Published:** 2024-01-18

**Authors:** Allan D. Wu, Benjamin L. Walter, Anne Brooks, Emily Buetow, Katherine Amodeo, Irene Richard, Kelly Mundth, Hooman Azmi

**Affiliations:** ^1^Parkinson’s Disease and Movement Disorders Center, Department of Neurology, Feinberg School of Medicine, Northwestern University, Chicago, IL, United States; ^2^Stanley Manne Children’s Research Institute, Ann & Robert H. Lurie Children’s Hospital of Chicago, Chicago, IL, United States; ^3^Center for Neurological Restoration, Department of Neurology, Neurological Institute, Cleveland ClinicCleveland, OH, United States; ^4^Parkinson’s Foundation, New York, NY, United States; ^5^Department of Neurology, Westchester Medical Center, Poughkeepsie, NY, United States; ^6^Department of Neurology, University of Rochester Medical Center, Rochester, NY, United States; ^7^Epic Systems Corporation, Verona, WI, United States; ^8^Department of Neurosurgery, Hackensack University Medical Center, Hackensack, NJ, United States; ^9^Hackensack Meridian School of Medicine, Nutley, NJ, United States

**Keywords:** electronic health record, Parkinson’s disease, hospitalization, safety, Epic Systems

## Abstract

Electronic Health Record (EHR) systems are often configured to address challenges and improve patient safety for persons with Parkinson’s disease (PWP). For example, EHR systems can help identify Parkinson’s disease (PD) patients across the hospital by flagging a patient’s diagnosis in their chart, preventing errors in medication and dosing through the use of clinical decision support, and supplementing staff education through care plans that provide step-by-step road maps for disease-based care of a specific patient population. However, most EHR-based solutions are locally developed and, thus, difficult to scale widely or apply uniformly across hospital systems. In 2020, the Parkinson’s Foundation, a national and international leader in PD research, education, and advocacy, and Epic, a leading EHR vendor with more than 35% market share in the United States, launched a partnership to reduce risks to hospitalized PWP using standardized EHR-based solutions. This article discusses that project which included leadership from physician informaticists, movement disorders specialists, hospital quality officers, the Parkinson’s Foundation and members of the Parkinson’s community. We describe the best practice solutions developed through this project. We highlight those that are currently available as standard defaults or options within the Epic EHR, discuss the successes and limitations of these solutions, and consider opportunities for scalability in environments beyond a single EHR vendor. The Parkinson’s Foundation and Epic launched a partnership to develop best practice solutions in the Epic EHR system to improve safety for PWP in the hospital. The goal of the partnership was to create the EHR tools that will have the greatest impact on outcomes for hospitalized PWP.

## Introduction

In 2009, the Health Information Technology for Economic and Clinical Health Act (HITECH) was passed into law to encourage hospitals to adopt electronic health record (EHR) systems. EHR systems can be configured to support significant improvements in patient care and quality by streamlining processes, consolidating availability/accessibility of information, and providing appropriate clinical decision support. Hospitals can leverage EHR systems to reduce safety risks for vulnerable populations, like people living with Parkinson’s disease (PWP).

PWP are hospitalized 1.5 times more frequently than their peers (Gerlach et al., 2011), with studies showing up to 45% of all people with Parkinson’s visiting the emergency department annually and up to 28% being admitted to the hospital (Oguh and Videnovic, 2012). During their hospitalization, PWP are more susceptible to hospital-acquired complications (Sauro et al., 2017), including worsening of their motor symptoms (Gerlach et al., 2012, 2013; Fujioka et al., 2016), delirium (Gerlach et al., 2012; Patel et al., 2017; Lawson et al., 2019), and dysphagia (Martinez-Ramirez et al., 2015), and are at increased risk for falls (Wedmann et al., 2019). In addition, they often have longer lengths of stay, are discharged to a facility rather than their home, and have higher readmission rates (Mahajan et al., 2016; Perdomo-Lampignano et al., 2020). The gaps in safety also increase the cost of care. In 2014, inpatient care for PWP (Mantri et al., 2019) was estimated to cost the Medicare program over $2.1 billion. Across all insurance types, PWP were estimated to contribute to over $7 billion in excess medical costs is for inpatient care (Lewin Group, 2019).

There are a number of reasons that PWP are at risk of experiencing hospital-acquired complications. First, patients are not always identified as having PD when admitted to the hospital. Second, because PWP are often not directly admitted for PD, appropriate management of their PD medications may not be a primary focus of care (Shin and Habermann, 2016). Finally, there is a PD knowledge gap in the healthcare workforce and the intricacies of managing Parkinson’s disease can be overshadowed by other more common disorders.

EHR systems are often configured to address these challenges and improve patient safety for PWP. For example, EHR systems can help identify PD patients across the hospital by flagging a patient’s diagnosis in their chart, preventing errors in medication and dosing through the use of clinical decision support, and supplementing staff education through care plans that provide step-by-step road maps for disease-based care of a specific patient population. However, most EHR-based solutions are locally developed and, thus, difficult to scale widely or apply uniformly across hospital systems.

In 2020, the Parkinson’s Foundation, a national and international leader in PD research, education, and advocacy, and Epic, a leading EHR vendor with more than 35% market share in the United States (Blauer and Warburton, 2023), launched a partnership to reduce risks to hospitalized PWP using standardized EHR-based solutions. This article discusses that project which included leadership from physician informaticists, movement disorders specialists, hospital quality officers, the Parkinson’s Foundation and members of the Parkinson’s community. We describe the best practice solutions developed through this project. We highlight those that are currently available as standard defaults or options within the Epic EHR, discuss the successes and limitations of these solutions, and consider opportunities for scalability in environments beyond a single EHR vendor.

## Methods

The Parkinson’s Foundation and Epic launched a partnership to develop best practice solutions in the Epic EHR system to improve safety for PWP in the hospital. The goal of the partnership was to create the EHR tools that will have the greatest impact on outcomes for hospitalized PWP and where there is the greatest likelihood for change in the inpatient setting.

### Collaborators: the Epic Movement Disorders Specialty Subcommittee

Parkinson’s Foundation is a nonprofit organization with a mission to make life better for people with PD by improving care and advancing research toward a cure. They are committed to leading the national effort to improve hospital care through systemic changes in areas of policy, technology, culture and education. Its Hospital Care Initiative aims to eliminate preventable harm and promote reliability in care for PWP in the hospital. The Parkinson’s Foundation Hospital Care Recommendations, released April 2023, (Parkinson's Foundation, 2023) outline five standards of care that represent optimal care in the hospital and can be used by hospital leadership to assess and systemically improve the quality of PD care within their institutions. The Recommendations were created in partnership with Hackensack Meridian Health, Henry Ford Health, and the University of Florida Health Norman Fixel Institute for Neurological Diseases, with support from Dr. Peter Pronovost and Manatt Health. The Hospital Care Initiative and Recommendations provided a national, patient-centered, practical starting point for developing the EHR-based solutions.

A Parkinson’s Foundation Ambassador, a volunteer living with PD who is specially trained on the topic of hospital safety, facilitated the connection between the Parkinson’s Foundation and Epic, which made this project possible. Additional Ambassadors were added to the subcommittee throughout the process as needed.

Epic (Epic Systems Corporation, Verona, WI) is an EHR software vendor with the largest percentage share of acute hospitals beds in the United States (Blauer and Warburton, 2023). Epic develops and maintains an out-of-the-box EHR system called the “Foundation System.” Hospital systems use the Foundation System to create their customized EHR, accounting for local workflow and operational requirements. Organizations using Epic can leverage the Foundation System as a vehicle to implement newly available and updated features developed by Epic (build packages).

To support the specific needs of specialty practices, Epic sponsors the Specialty Steering Boards (SSB), consisting of subject-matter experts who advise Epic on best practice workflows, default content, and ideal EHR features beneficial for each specialty. All SSB members are peer-selected volunteers from that specialty. The Adult Neurology Specialty Steering Board (Neurology SSB) was formed in 2016. The collaborative and productive nature of the Neurology SSB, in partnership with advocates within the American Academy of Neurology, has been previously described (Weathers et al., 2019).

The Epic Movement Disorders subcommittee (EMDS) was formed to develop, recommend, and promote Epic EHR solutions to reduce risks to hospitalized PWP. The EMDS initially included select members of the Neurology SSB and representatives from the Parkinson’s Foundation including staff leadership and Parkinson’s Foundation Ambassadors. Additional subject matter experts were identified and added to the EMDS during the review of possible EHR solutions. An Epic facilitator with a neurology nursing background coordinated efforts as the project manager.

### Approach and roadmap

#### Initial state and locally developed solutions

In 2020, the Epic Foundation System had no default features that would specifically reduce risks of inappropriate medication dosing or timing. As such, any existing solutions using the Epic EHR were locally developed by individual hospital systems. The EMDS sought examples of such solutions from hospitals in the Parkinson’s Foundation Global Care Network, known national leaders in inpatient Parkinson’s care, and Neurology SSB contacts. A series of demonstrations were conducted to review possible solutions.

#### Roadmap and implementation

A review of existing solutions and discussions with multiple subject-matter experts identified both opportunities for leveraging existing Epic EHR features and highlighted gaps in the existing Epic EHR feature set that limited the development of some proposed solutions. A roadmap was devised, leveraging the best solutions, and balancing feasibility, usability, and clinical prioritization. Through a series of development cycles, the EMDS identified, prioritized, and then designed and built best practice content and workflows that are now available as default build packages in the Epic Foundation System.

## Results

### Review of locally developed solutions

Hackensack University Medical Center (HUMC), Northwestern University, Cleveland Clinic, and the University of Rochester all developed individual solutions to improve safety for hospitalized PWP using the Epic EHR. Presentations of these solutions led to productive discussions and ideas within the EMDS. Key features from each solution considered for the roadmap are summarized in Table 1.

**Table 1:** Locally developed solutions reducing risks to hospitalized persons with Parkinson’s disease.

Target areas for best practice solutions	Identification of hospitalized PWP	Prevent medication errors through medication order entry customization	Care plan for inpatient nursing/support staff
Hackensack University Medical Center (HUMC)	Flags and banners in Chart Review	Default and custom frequency timing for levodopa medication orders.	Care plan integrated as Epic Nursing Care Plan
University of Rochester Medical Center	Best Practice Advisory (avoid neuroleptics)		
Cleveland Clinic	Patient List dashboard	Default and custom frequency timing for levodopa medication orders. Custom After-Visit Summary.	Protocol screening for PD-related medication errors or complications
Northwestern Medicine	Best Practice Advisory (suggest PD Order Set)	Default and custom frequency timing for levodopa medication orders. Custom PD Order set.	Inpatient medication reconciliation nursing in-service training

PWP, Persons with Parkinson’s disease; PD, Parkinson’s disease; Custom time-based.

### Hackensack University Medical Center

**Figure 1:**
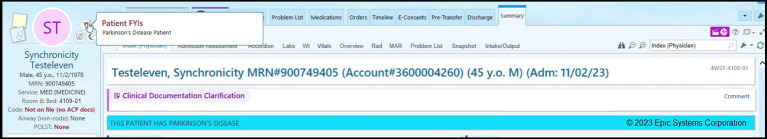
Patient identification tools alerting users of a person with Parkinson’s disease. Screenshot showing EHR Chart (HUMC) for hospitalized PWP. Parkinson’s Disease is highlighted as a banner (blue).

Proper identification of patients is crucial for successful implementation of any protocol. Hackensack University Medical Center (HUMC), with the help of the technology team, developed flags that would alert any provider to the diagnosis of PD when a chart was opened. The flags appear as an icon in the Storyboard and as a banner across the summary report in the chart (Figure 1). The triggers for the flag are the inclusion of PD in the problem list, as well as detection of the keywords including “Parkinson” in any note. While this does result in the inclusion of atypical parkinsonism syndromes, as well as occasional patients that may not have PD, the increased sensitivity was felt important to avoid erroneously missing potential patients in the ensuring protocol.

**Figure 2:**
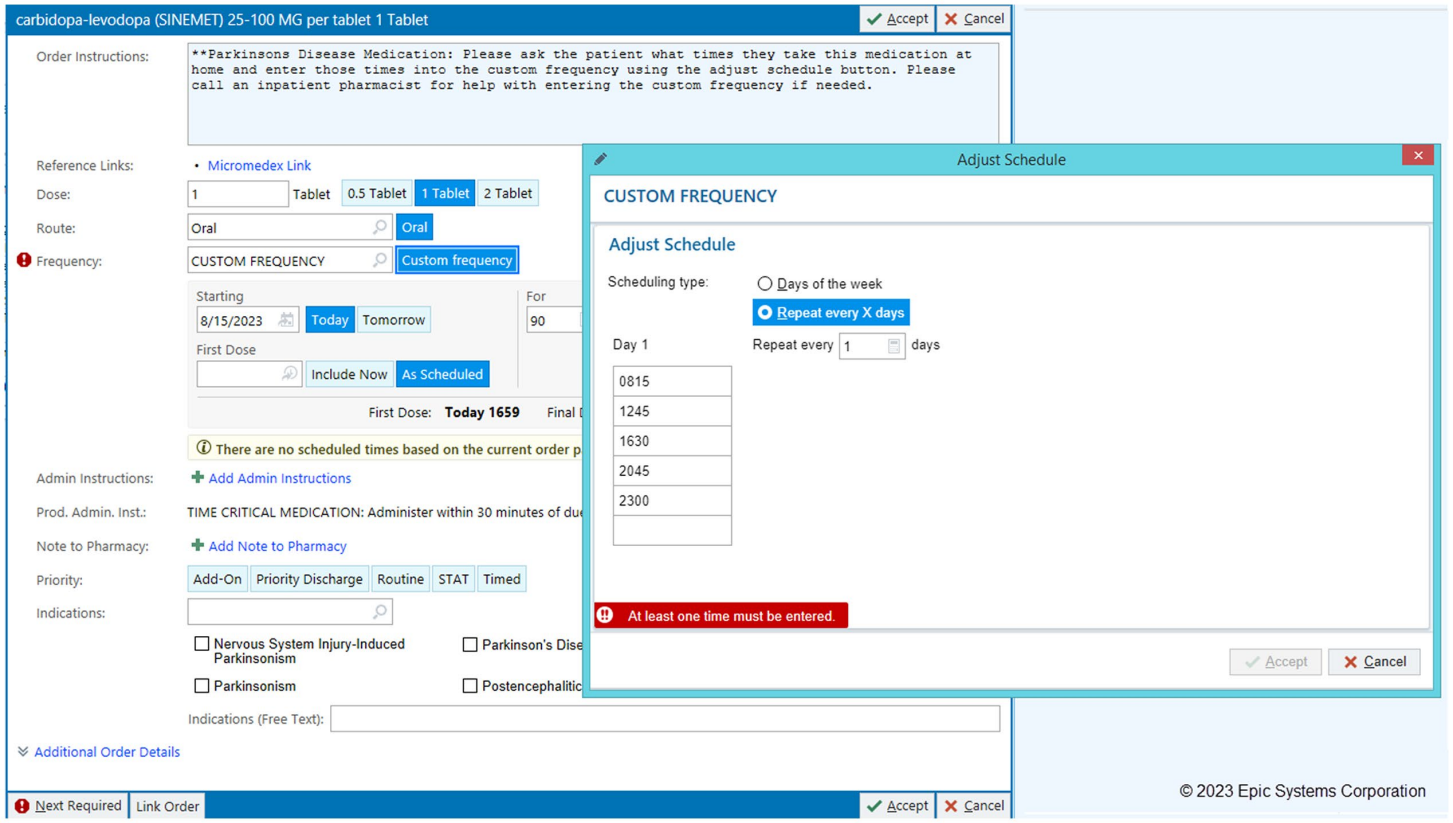
Examples of default and custom frequency timing for inpatient levodopa medication orders custom frequency created for levodopa medication, eliminating standard non-custom options, and requiring timing of medication to be entered (HUMC).

A protocol for ensuring adherence to the home schedule and timing for PD meds was developed at HUMC with an emphasis on using customized time-based levodopa medication orders. Initially, and common to all institutions, the default options available when ordering PD medications in the inpatient setting were standard default frequency settings (once a day, nightly, twice, 3x, or 4x per day). These standard frequency settings would result in default hospital timings that were rarely aligned with actual timing of PD medications. To order the medication in a custom format, providers would have to follow a three step process: (1) avoid selecting the default, (2) click a search button and search for a particular option called “custom frequency” in the drop-down menu, and after selecting it, (3) insert the appropriate timing for the medication. In addition to the paucity of knowledge about the importance of custom ordering for PD medications, these extra steps were additional barriers to placing appropriate orders for PD medications. The HUMC team was able to successfully add the “Custom” option as a default frequency for selected PD medications for which timing is critical and eliminate the default frequency settings (non-custom) options from being available to choose (Figure 2). With these changes, HUMC saw nearly a threefold increase in appropriate (custom) placement of PD medication orders (Azmi et al., 2020).

**Figure 3:**
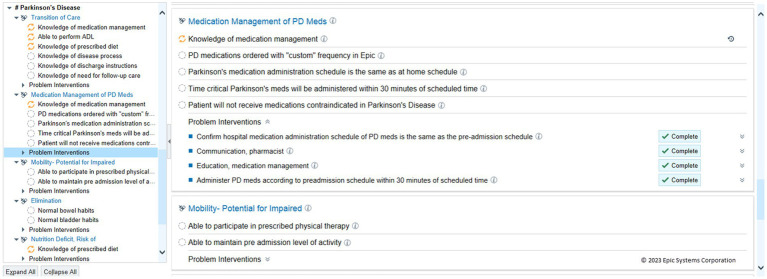
Nursing care plan for hospitalized patients with Parkinson’s disease. Care Plan for patients with Parkinson’s disease regardless of admitting diagnosis (HUMC).

Care plans are available options within the Epic EHR for nursing teams to ensure adherence to required standards or locally desired guidelines or protocols. They are step-by-step road maps for care of a specific patient population. Many have been used for older adults, including those specifically with cognitive decline, diabetes, and other complex and long-term needs (Yutong et al., 2023). streamlining care and ensuring adherence to standards. A nursing PD care plan was developed at HUMC to supplement and support our educational efforts to reduce risk for hospitalized PWP. The care plan can be added by nursing intake for any PWP admitted. In addition to existing hospital-wide components of mobility and fall prevention, our care plan incorporates our goals of adherence to patients’ home medication regimen, placement of orders for PD medications in custom format, the time-critical nature of PD medications, and avoidance of administration of contraindicated medications. While the use of care plans at HUMC are not required, they are encouraged (Figure 3).

### Northwestern University

**Figure 4:**
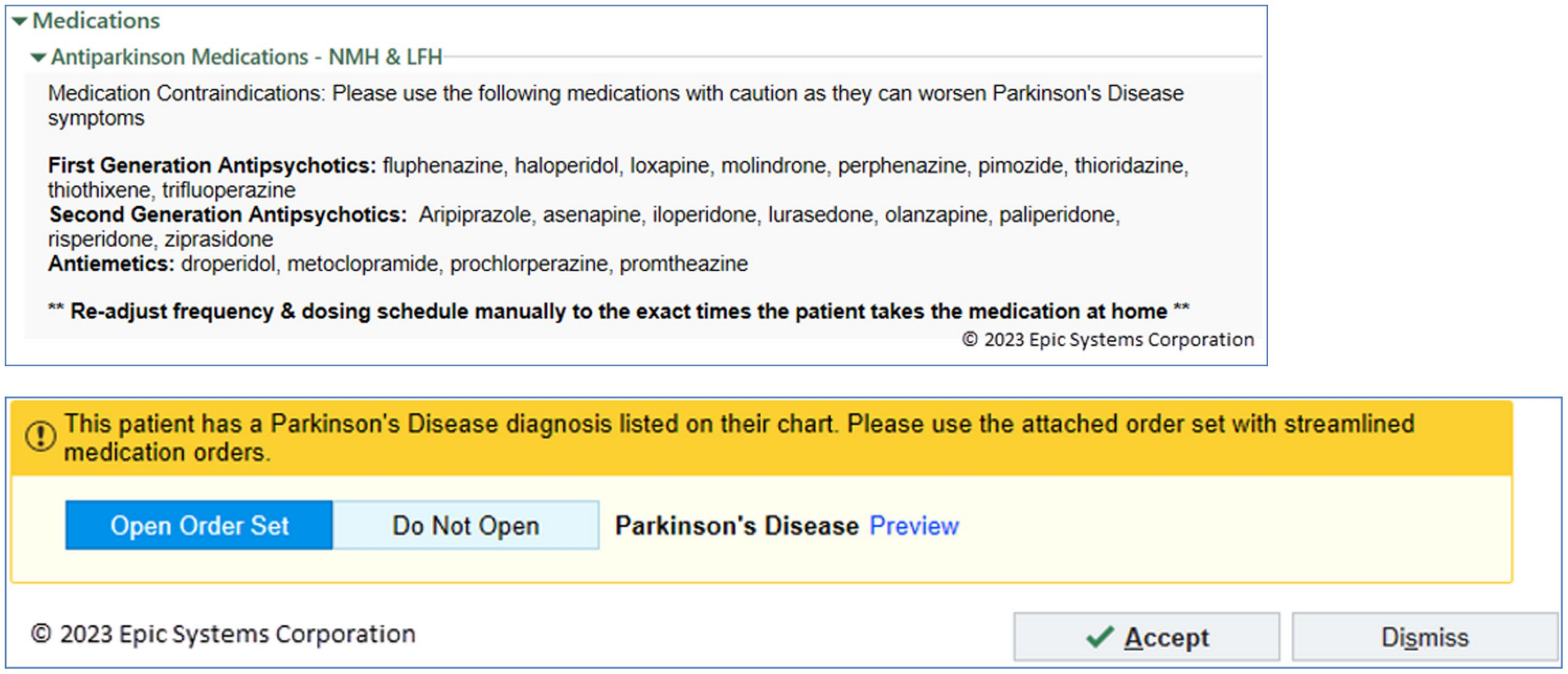
Parkinson’s Disease Order Set. (Top) PD Order Set with text advisory to avoid contraindicated medications and recommendation to ensure PD medications using exact frequency and dosing (Northwestern); (Bottom) Associated Best Practice Advisory (BPA) Alert, supporting ability to open the PD Order set with a single click (Northwestern).

To address risks of medication errors in the hospital, Northwestern’s Movement Disorders team, within the Parkinson’s Foundation Center of Excellence, developed a PD-specific Inpatient Order Set, which included prominent advisory text to address two issues. First, the advisory text prominently warned of the need to avoid relatively contraindicated neuroleptic medications (Figure 4A). Second, advisory text noted that certain PD medications, particularly levodopa medications, would require manual entry of exact frequency and dosing schedules as best practice. All inpatient medication orders of levodopa, similar to HUMC and other institutions, had default medication frequencies removed. A ‘User Specified’ custom default frequency was provided for each levodopa medication order which would then require discrete entry of timings for the medication. This would occur if the medication was ordered within or outside of the order set.

The major limitation was awareness and use of this custom PD Order Set. A best practice advisory (BPA) alert was created to detect the diagnosis or the presence of Parkinson’s disease on the problem list (Figure 4B). It would suggest, with one click, the ability to open the PD Order Set with its guidance and suggested PD medication timings and dosings. After releasing this BPA, 33% of PWP who were hospitalized had providers open the PD Order Set, representing the opportunity to see the guidance for ordering inpatient PD medications.

Another limitation identified was that the nursing staff who perform medication reconciliation intake in a prior to admission home medication workflow are unable to access the dosing/timing table required when ordering PD medications with detailed ‘User Specified’ doses and timings. These nursing staff are in the best position in the workflow to identify exact doses and timing during the intake/admission process. This represents a fundamental limitation in the EHR design in that home medications do not support exact doses or timings that can translate to the inpatient setting. As such, there is an inability to efficiently transfer that outpatient PD doses/frequency information to the appropriate inpatient ordering clinician. A quality improvement process involving nursing informatics designed a workflow where the home medication doses and timing were placed in the comment field, and the clinicians who then continue and order that medication into the hospital encounter are asked to transcribe the free text Comment information into the ‘User Specified’ table during medication ordering. This workflow was poorly utilized by nursing staff and admitting physicians. The standard workflow requires nursing staff to routinely identify outpatient medications that the patient is taking and then to enter (or reconcile) each medication into the inpatient EHR upon admission. This workflow required the nursing staff to recognize that a medication was PD-related and to enter exact times of day that patient took that medication as outpatient into a free text field. Then, admitting physicians, when writing inpatient orders, were required to remember to look at that free text field for those PD particular medications, and translate those free text notes into discrete exact times into a PD medication order to sign. This workflow imposes extra burden on both nurses and physicians. In spite of promotional materials and tipsheets, this awkward workflow was rarely used and reflects current difficulties of communicating key medication timing information between nurses and physicians within the EHR.

### Cleveland Clinic

**Figure 5:**
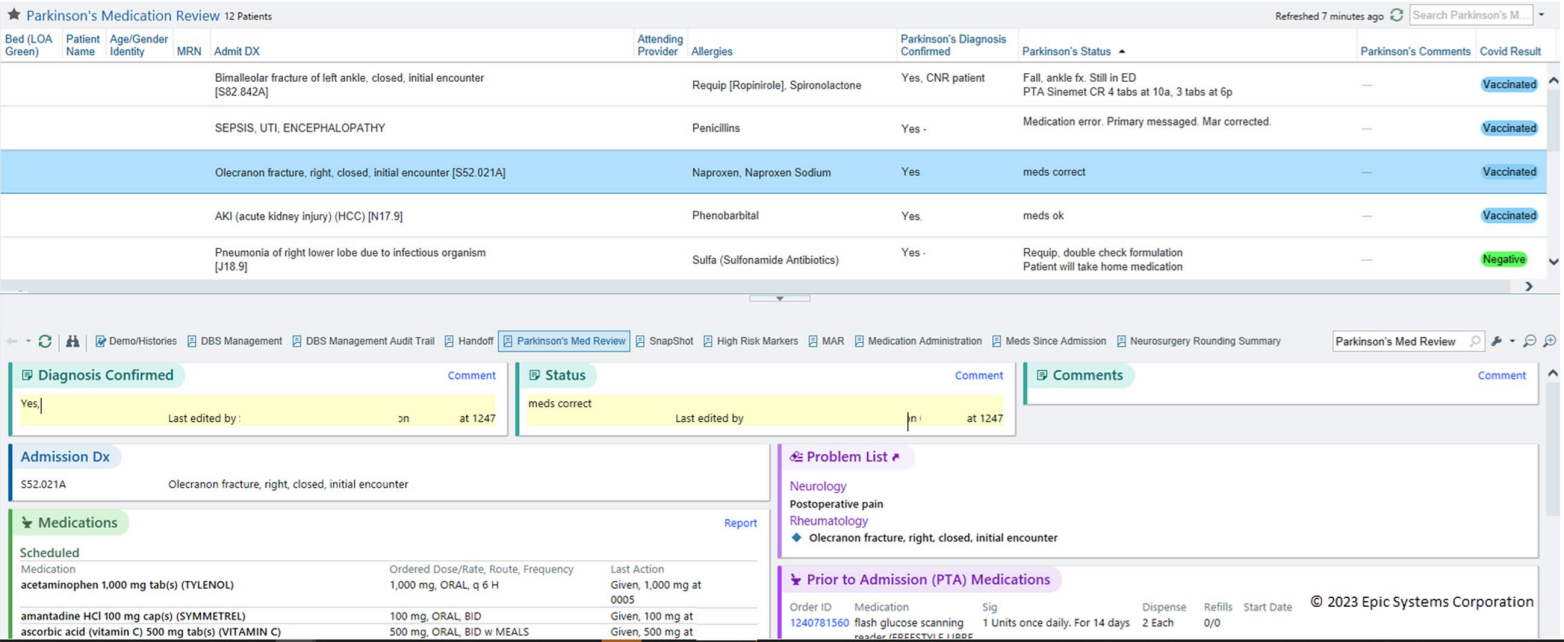
Patient List of PWP with monitoring columns and summary report. Real-time System List of inpatients admitted with PD, monitored by a safety team. An interactive summary report provides key information about the selected patient, and includes areas for comments and status updates that update the columns shown in the Patient List (Cleveland Clinic).

The Cleveland Clinic, a Parkinson’s Foundation Center of Excellence, developed a proactive strategy to improve inpatient care for people with PD. This strategy utilizes a team of advanced practice providers from the outpatient movement disorder clinic and heavily leverages IT strategies through the Epic EHR. Using a cross-population approach, Cleveland Clinic developed an automated Patient List which populates in real-time with all hospitalized patients with a diagnosis, past medical history, or problem list identifying PD or parkinsonism (Figure 5). Advanced Practice Providers review this list of patients daily, looking for probable errors in medication orders that do not match home regimens, either based on available EHR outpatient documentation or on patients and caregivers record of the home regimen (if not documented within the EHR). This review also looks for evidence of PD-related symptom exacerbation related to acute hospitalization. An associated custom report displays relevant information and allows comments and status information to be updated and shared with care teams.

A key advantage of this strategy is the additional benefit of skilled PD clinicians ensuring quality care for all people with PD who are admitted. As these skilled clinicians utilize this list they observe the effects of implementation in real time, identifying opportunities to improve clinician-lead as well as EHR-driven quality assurance practices.

Many tools mentioned in this paper are also used, including contraindicated medication alerts and constraints to custom time-based levodopa medication orders. However, additional advice can be given, and further care gaps are frequently identified as we proceed with this quality improvement program. With the collaboration and support of a Parkinson’s Foundation grant, the impact of this approach is being studied and compared to a baseline data set in which we could define the frequency of medication timing deviations and contraindicated medications (Yu et al., 2023).

### University of Rochester Medical Center

The University of Rochester Movement Disorders Parkinson’s Foundation Center of Excellence, with support from a Parkinson’s Foundation Community Grant, assembled a team of PD Champions, including neurologists, hospitalists, physical therapists, occupational therapists, speech therapists, and registered nurses at Highland Hospital in Rochester, NY. As part of the initiatives driven by the team, collaboration between inpatient pharmacists, Epic EHR team developers, and neurologists led to the creation and implementation of a best practice advisory (BPA) to define the acronym warning against the administration of dopamine-blocking agents (i.e., antipsychotics and certain antiemetics) to PWP and related parkinsonian disorders. The implementation of the BPA started with a meeting among the BPA committee to discuss the harm associated with exposing PWP to a dopamine-blocking agent. The team then created an inclusive BPA that would be triggered should any antipsychotic other than quetiapine, pimavanserin, or clozapine be ordered on a patient with diagnoses (using linked ICD-10 codes) of either PD, multiple systems atrophy, corticobasal degeneration, dementia with Lewy bodies, or progressive supranuclear palsy. The final push with the BPA “going live” was to demonstrate its need. This was accomplished by showing the BPA was triggered ten times over a one-month period in which the BPA was only visible to the inpatient pharmacy. This was then brought to the BPA committee, and the BPA was approved and went “live.” Ongoing data collection and analysis to demonstrate its effectiveness are underway.

### A roadmap and Epic Foundation System

The EMDS identified seven workflows that are essential in reducing safety risks for PWP in the hospital based on a review of the existing literature, the example solutions described above, and care recommendations from the Parkinson’s Foundation Hospital Care Initiative. The EMDS focused on developing EHR solutions that could become default features in the Epic Foundation System. A summary of these workflows and associated solutions is shown in Table 2. Many of the solutions described are currently available to Epic users and some remain in active development. Each organization upgrades their instance of Epic to a given dated version on its own schedule. Table 2 indicates which features are available now with the associated version of Epic; which features are being built by Epic for an upcoming release; and which features are on the roadmap for future releases. Features listed as available can be used or turned on without incurring costs for development. All features have default configurations set, and most organizations should review defaults to determine the best way to activate the feature and promote its actual use within local workflows.

**Table 2:** Roadmap for tools to reduce risk for hospitalized PWP in the Epic Foundation System.

Roadmap workflows	Epic EHR-based solution	Epic Foundation System Status
Identify admitted PWP and their unique care needs	Storyboard icon and report with guidelines and link to Parkinson Foundation resources. Patient List columns for real-time monitoring of neurology, PT, OT, SLP consults on admitted PWP. Reports summarizing inpatients with PT, OT, SLP referrals and both home & inpatient PD-related medications. Reports to identify PWP who are current admitted, recently discharged, and have upcoming pre-admissions.	Live since Epic February 2020 version. Live since Epic February 2020 version. Live since Epic February 2020 version. Live since Epic November 2022 version.
Notify patient’s neurologist when PWP is admitted	New Care Team relationship of neurologist available to allow users to configure notifications to neurologists of admitted PWP	Live since Epic November 2022 version.
Prevent administration of contraindicated meds to PWP	Drug-disease warnings from third party medication vendors (PD-med warnings available) Best Practice Advisory (alert) to supplement drug-disease warnings. Comprehensive medication alerts and warnings for PWP	Live since Epic 2009 version. Design in progress (not live) Specialty organizations to address with third party medication vendors
Protect PWP home med regimen during hospital stay	Ability to prescribe outpatient medications to a discrete timed schedule. Updates to PD medications to allow outpatient discrete timing frequencies to be entered discretely into inpatient PD medication timing; updates to orders, preference lists, order sets; prevent PD medications from defaulting to inpatient pharmacy administration times.	Epic development enhancement submitted. Design pending (not live).
Enforce timely administration of PWP’ PD meds during admission	Add logic to consider PWP medications overdue 15 min after due time. Reports to audit timeliness of medication administrations. Report of PD-related medication administrations more than 15 min after due time.	Design pending (not live). Live since Epic 2014 version. Design pending (not live).
Document and display a PWP Nursing Plan of Care	Develop a Nursing Plan of Care for PWP (based on HUMC content).	Design pending (not live).
Improve discharge planning and discharge instructions for PWP	Develop default workflow, content, and discharge instructions for PWP (based on Cleveland Clinic content).	Design pending (not live).

Roadmap workflows indicate EMDS identified priority workflows to be addressed in electronic health records. Epic EHR-based solutions indicate Epic-vendor specific tools and workflows recommended to address each roadmap workflow. Epic Foundation System Status indicates whether the solution is a feature already released for organizations to implement (and the earliest available version when that feature was available). Features may be available by default, require enabling or configuration, require a minimum version, or require update packages. Features are best paired with appropriate institutional workflow. Organizations interested learning more or enabling Epic Foundation features should contact their organization’s Epic representative for details. PT, physical therapy; OT, occupational therapy; SLP, speech/language pathology.

#### Identify admitted PWP and their unique care needs

**Figure 6:**
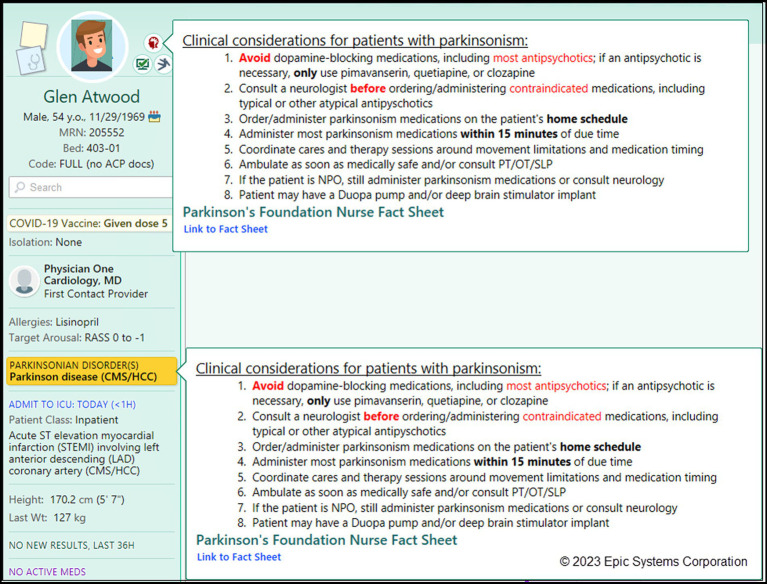
Epic Foundation System Storyboard icon and tooltip. A Storyboard icon appears when patients are admitted and have either Parkinson’s disease or a related parkinsonism disorder. The tooltip shown will automatically appear when the mouse hovers over either icon or problem list item. Users can click the Link to Fact Sheet hyperlink to directly open the Parkinson Foundation Fact Sheet. (Epic).

**Figure 7:**
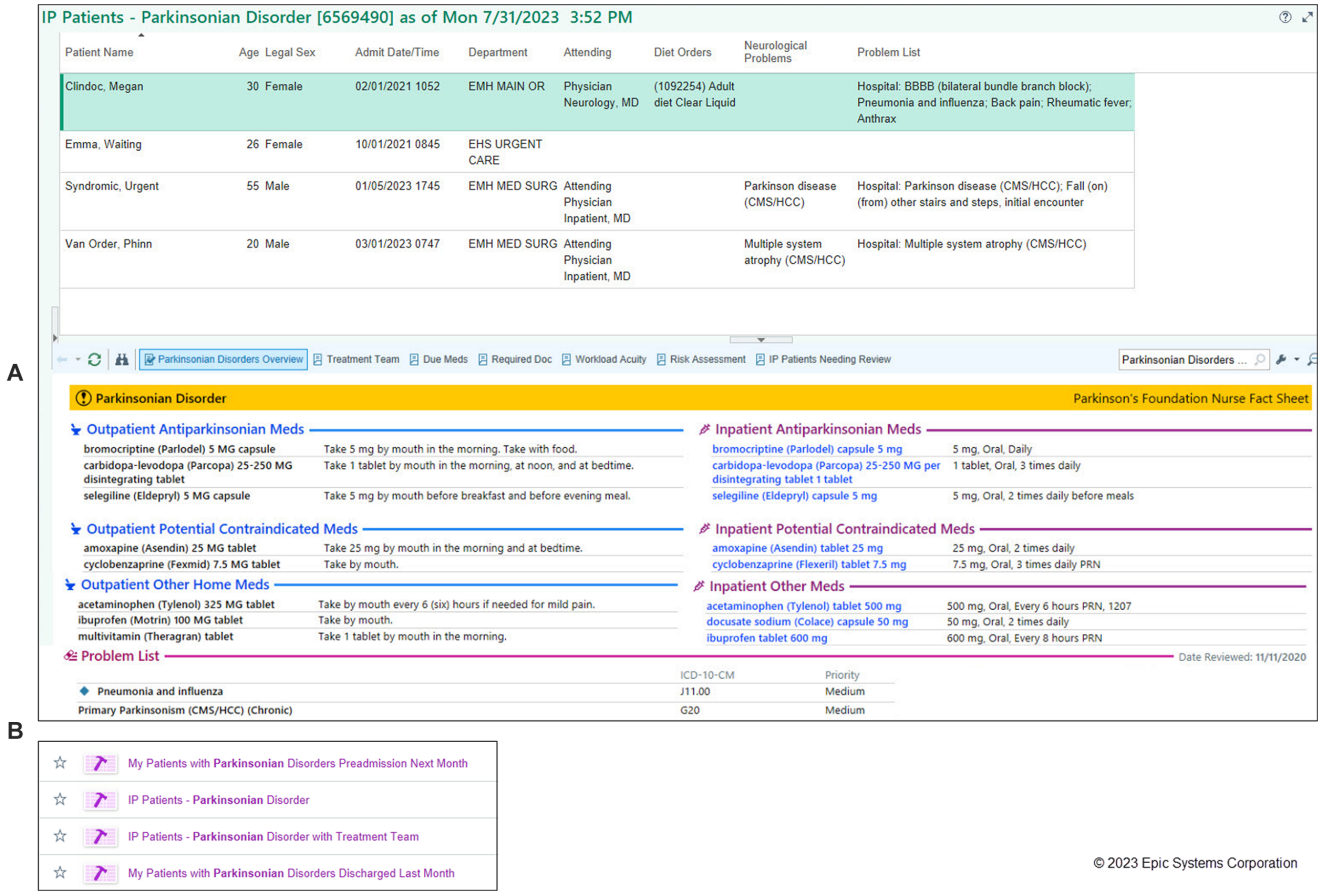
Epic Foundation System reports to find hospitalized PWP. Default available Reporting Workbench reports allow ad hoc searches of hospitalized PWP and related parkinsonism syndromes. **(A)** Default report shows relevant outpatient and inpatient medications by category with potential contraindications noted. Additional columns are available in Foundation to add to the report including whether consults to neurology, PT, OT, or SLP services have been ordered (not shown). PT, physical therapy; OT, occupational therapy; SLP, speech/language pathology. (Epic). **(B)** Report on patient status (in-patient, pre-admissions, discharged) which can be customized to institutional needs (Epic).

Starting in February 2020, organizations can implement an Epic-provided build package to add three tools:

1. A default Storyboard icon and banner to quickly see that a patient has a parkinsonian disorder. The Storyboard is a default sidebar where all relevant information about a given patient is prioritized with those of most importance near the top. Examples of Storyboard information include icons representing care teams, demographics, allergies, problem list, medications. Hovering over the icon or banner displays a tooltip summary of best practice care guidelines for inpatient care and a hyperlink to the Parkinson’s Foundation supporting documentation. Out-of-the-box, these notifications are only visible to neurologists, but organizations can and should consider configuring this feature, so it is visible to all inpatient users’ workspaces. Instructions on how to do so are included in the build package documentation (Figure 6).
  The icon appears based on a group of PD and parkinsonism diagnosis codes. A default grouper is provided in the Foundation System and can be leveraged by organizations to drive any custom PD-related content, including local solutions as previously described. Using (or updating to use a Foundation grouper) provides standardization for identifying PWP and related parkinsonisms.

2. An “IP [Inpatient] Parkinsonian Patients” report identifies hospitalized PWP and displays a summary of key clinical information important to caring for a PWP. This includes a comparison view of the patient’s outpatient prescriptions against the current inpatient orders and a call-out section of potentially contraindicated medication orders. Out-of-the-box this report is a default for neurologists in the Patient List and Summary activities and available to all users to add independently (Figure 7A).
3. Four new Patient List and Reporting Workbench columns help consulting neurologists, care teams, and PD safety teams see at-a-glance if the PWP has appropriate supportive orders in place. These columns indicate whether consults to neurology, physical therapy, occupational therapy, and speech therapy have been ordered during the current admission: “Neurology Service Consulted?,” “PT Consulted?,” “OT Consulted?,” “SLP Consulted?” Out-of-the-box, these columns are available to all users who choose to add them to their My Lists.

In November 2022, additional tools were added to the build package:

4. Additional reports to find PWP who are currently admitted for a given care team (“IP Patients - Parkinsonian Disorder with Treatment Team”), who were recently discharged (“Patients with Parkinsonian Disorders Discharged Last Month”) or have upcoming pre-admissions (“Patients with Parkinsonian Disorders Preadmission Next Month”). Out-of-the-box, these reports are available to all clinical users and can be configured to meet organizational or individual user needs or workflows (Figure 7B).

#### Notify a patient’s neurologist when PWP are admitted

**Figure 8:**
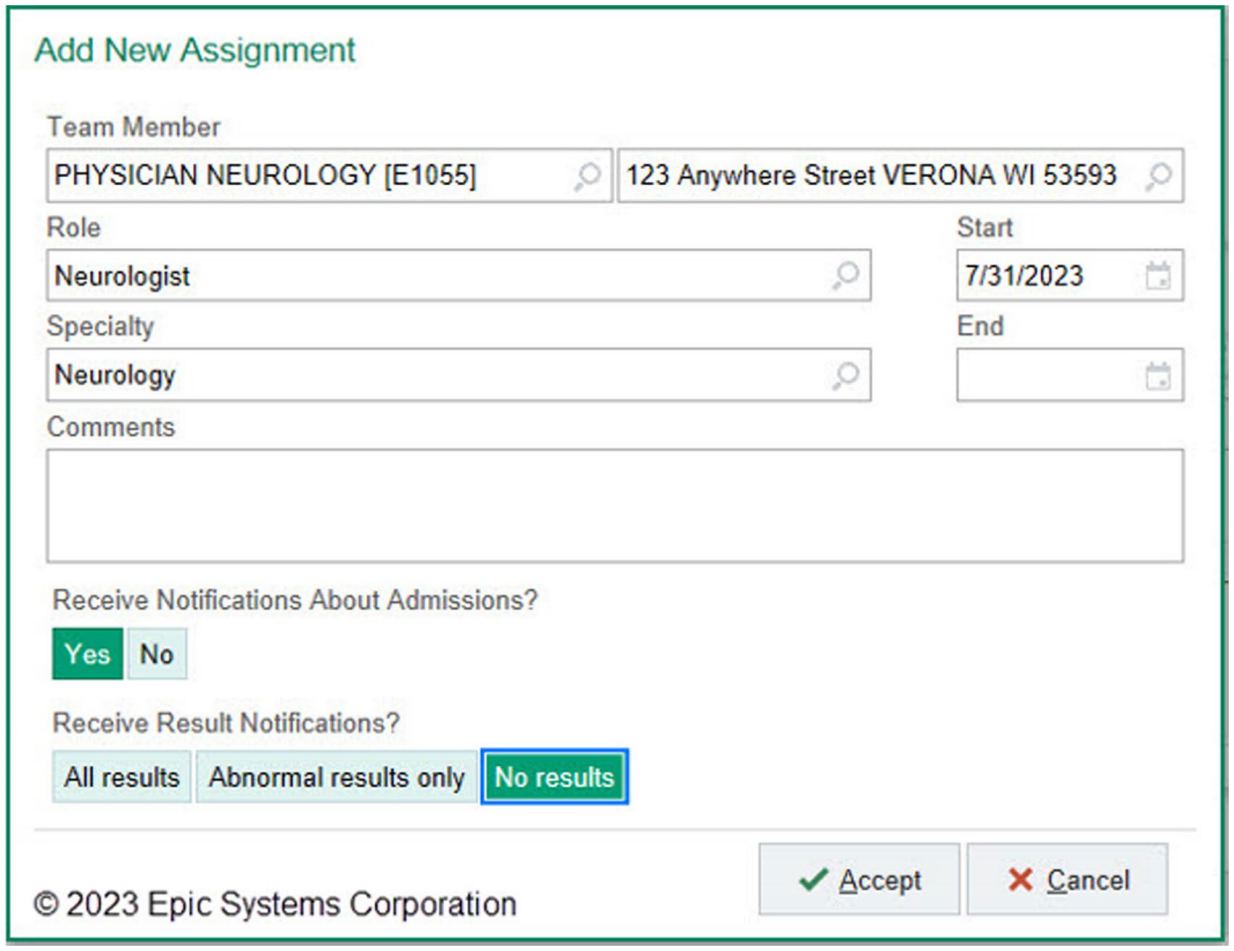
Epic Foundation System neurologist Care Team relationship. New Care Team relationship for “neurologist” allows providers to configure their admission notifications. Although admission notification is a standard feature in Epic, the ability to selectively notify neurologists was not previously present. (Epic).

In November 2022, Epic Foundation System added a care team relationship specifically for neurologists. This allows a provider to be listed as the PWP’s neurologist. Users can then choose whether to receive patient admission alerts, either by their defined relationship or on a patient-by-patient basis (Figure 8).

#### Prevent administration of contraindicated medications to PWP

**Figure 9:**
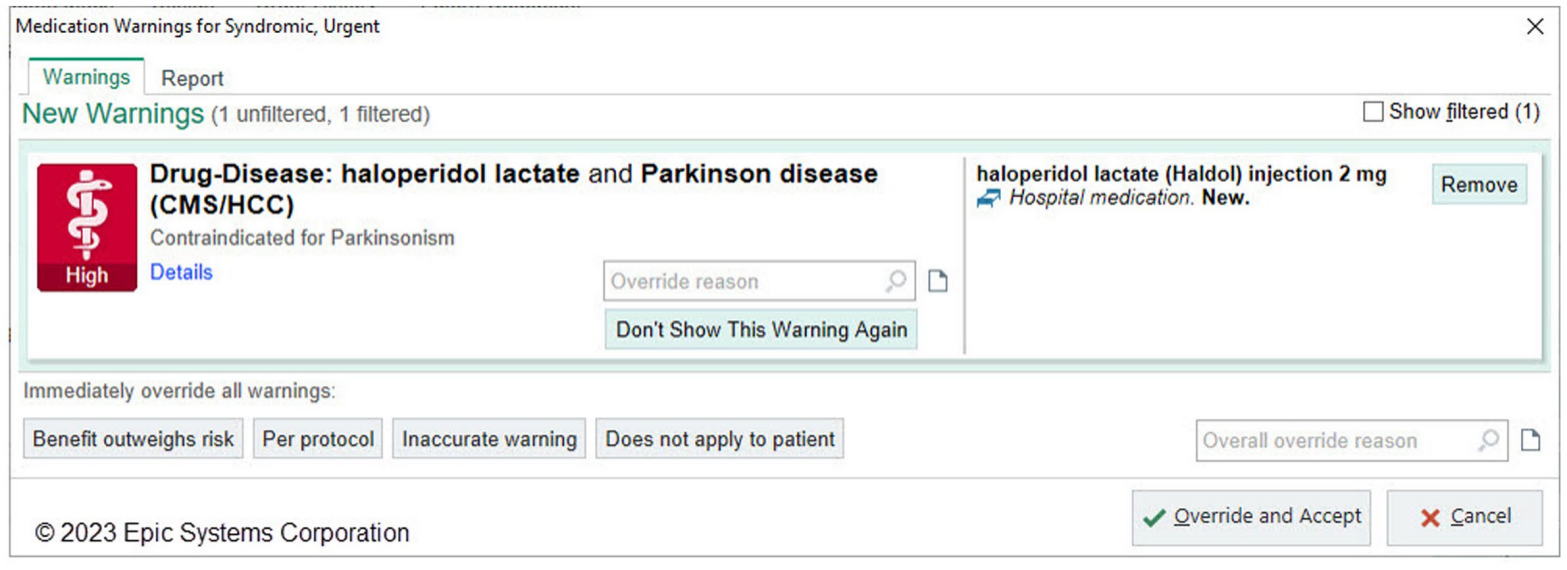
Epic Foundation System example alert of drug-disease clinical decision support alert. The Epic Foundation System includes drug-disease warnings loaded from different third-party medication vendors. This is an example of a warning that shows when the provider attempts to order haloperidol for a PWP. (Epic).

Drug-disease warnings are clinical decision support tools that can reduce risks of prescribing relatively contraindicated medications (Bhakta et al., 2019). The Epic Foundation System will include an example of a contraindication with haloperidol, a prototype typical neuroleptic and Parkinson’s disease (Figure 9). However, an effective BPA would ideally address broader classes of neuroleptic medications with varying degrees of risk.

Drug-related clinical decision support tools in EHRs are provided by third-party medication vendors and include interactions between drugs and drug-disease combinations. These interactions are imported into an organization’s local Epic instance and enabled by the local organization to display. After discussion, including the option to adapt the neuroleptic Best Practice Advisory developed by University of Rochester into Foundation System, the current Epic-focused development roadmap does not include a more comprehensive BPA due to challenges in maintenance and lack of consensus on categorization of interaction severity. The EMDS recommends that organizations enable, using standard Epic functionality, at minimum, Parkinson’s disease-drug related warnings from third-party interaction warnings labeled with a severity of “contraindicated” (Figure 9).

#### Protect PWP’ home medication regimen during the hospital stay

Unlike inpatient medication orders, although PWP usually take PD medications at stated times throughout the day, within most EHRs, outpatient medications cannot be prescribed at discrete, specific times. As a result, outpatient communication of precise timing of PD medications is in free text comments in the prescription or in separate instructions given directly to patients. The translation of outpatient frequency timing of these medications to the inpatient medication order once a PWP is hospitalized remains a significant gap in safety efforts. EMDS review of individual organizational solutions (Table 1) illustrates a variety of partial solutions including customization of medication frequency, default medication preference list, and order set updates to help neurologists order PWP home medications in a way that prevents inpatient admitting providers from ignoring the schedule. The EMDS identified this gap as a priority. As a result, Epic development of new medication functionality addressing this gap is on the road map as a current enhancement under design and development with EMDS and Epic Neurology Steering Board input. This widely anticipated feature will streamline inpatient admission medication reconciliation to allow direct continuation of discrete timing of PD home medication when PWP are hospitalized.

#### Enforce timely administration of PWP’ medications during hospitalization

The Parkinson Foundation Quality Initiative recommends that all parkinsonian-related medications are administered within 15 minutes of their due time. In the Epic Foundation System, inpatient medication orders are scheduled to discrete due times. By default any medication is considered overdue 60 minutes after the scheduled due time, but organizations can customize this time frame for groups of medications. Organizations can monitor medication administration compliance using out-of-the-box analytic tools. EMDS advises setting PD-related medications to be overdue 15 minutes after the scheduled due time and plans to design more robust analytic tools specific to monitoring timeliness of PWP medication administrations in a future release.

#### Document and display the PWP’s nursing plan of care

The subcommittee plans to review and adapt the nursing care plan from HUMC into Epic’s Foundation System in a future release.

#### Improve discharge planning and discharge instructions for PWP

The subcommittee plans to design workflows and content to improve discharge planning and discharge patient instructions for PWP based on solutions from Cleveland Clinic. In addition, the planned development of an Epic enhancement that supports outpatient medication timing frequency will also support discharge safety by allowing translation of an inpatient medication timing to an outpatient prescription at discharge.

## Discussion

Parkinson’s disease (PD) patients are commonly recognized to be at risk for deterioration following hospitalization. In a recent comprehensive literature review, Gerlach et al., 2011 reported that PD patients appear to be at both a greater risk for hospitalization with estimations in the range of 7–28% per year, about 1.5 times the general population, and also tend to be admitted for a longer inpatient stay by 2–14 days. This review highlighted the range of issues identified and noted that few systematic solutions for preventing adverse outcomes in this patient population have been identified. This review also indicated that existing literature focused chiefly on perioperative deterioration and emergency room visits and pointed out potential avenues for research, including a better identification of risks involved with hospitalization. In their review, they also found a 31% dissatisfaction rate of PD patients concerning the inpatient management of their condition. A follow-up study by the same group reported results from a survey of 684 PD patients and found that 18% of PD patients were hospitalized within a year (Gerlach et al., 2012). The study reported that 21% of hospitalized PD patients noted deterioration of motor symptoms after hospitalization, 33% had complications during hospitalization, and 26% reported incorrect medication management. In addition, patients with a higher levodopa equivalent dose (LED) of PD medications were associated with an increased risk of post-hospitalization deterioration of PD function.

This report illustrates an approach to scalability to address these issues, albeit limited to Epic Systems, a single major EHR vendor. The Epic Movement Disorders Subcommittee, a collaboration between Epic and the Parkinson’s Foundation, worked to develop EHR-based best practice solutions that could become standard within Epic’s Foundation System as either default or as available features to address challenges faced by PWP in the hospital. The Parkinson’s Foundation contributed a national and patient-oriented perspective and a toolkit to reduce these hospitalization risks. This report contributes to addressing the gap between the resources in the Parkinson’s Foundation toolkit and the availability of EHR-based solutions that can be deployed with routine updates to all customers of Epic.

As a vendor, Epic releases many clinical programs, published as implementation and workflow guides that often include specific features built into Foundation System. Examples include tools for sepsis screening, opioid monitoring, fall risk screening, advance care planning documentation, infant feeding best practices, and delirium prevention in ICUs. Most programs are in response to regulatory or certification incentives or requirements. The EDMS plans to create a clinical program publication highlighting the workflows and solutions described in this article.

This report highlights two types of solutions. First, we illustrate a consensus roadmap for development and highlight released features in Epic EHR systems based on this EMDS collaboration. Features released in this roadmap have the advantage of becoming standard within the Epic toolkit, being aligned with a national PD safety initiative, and being deployed automatically via routine Epic upgrades. While a standard toolkit within an EHR can provide features available for implementation and enhance standardization across customer organizations, local configuration and customization are often still needed. Thus, this report also presents examples of practical solutions from different organizations, which can serve readers as further starting points for implementation of features not yet available in the toolkit.

The overall EMDS essential workflows for improving patient safety can be grouped into three categories: identifying admitted PWP, medication management, and PD care plans.

### Identify admitted PWP

Though PWP are overall admitted to the hospital more often than peers, there is still limited awareness of the frequency of the hospital care provided to this population due to the fragmented nature of their admissions. One systematic review reports the percentage of admissions due to various concerns as 22.0% infections (primarily urinary tract infection and pneumonia), 19.0% motor symptoms, 18.0% falls and fractures, 13.0% cardiovascular comorbidities, 8.0% neuropsychiatric disorders, and 7.0% gastrointestinal disorders (Okunoye et al., 2020). One can assume that patients entering the hospital for these reasons would be spread across nearly every hospital unit.

Disease-specific real-time patient lists, reports, or dashboards of PWP can be robust tools for population-based interventions or care paradigms once PWP are hospitalized. The Epic Foundation System now contains multiple tools out-of-the-box that support identification of PWP automatically (Storyboard flags and banners), by real-time lists (Patient Lists), or by pre-configured reports and dashboards. These tools enable local organizations to develop and prioritize approaches that can lead to sustainable novel care models that focus on specialized resources for vulnerable patients and, in this case, brings providers that are typically outpatient-focused to the inpatient arena, where most complications occur. As Epic (and other vendors) develop more support for out-of-box alert features for various conditions beyond PD, rather than just turning on all of them, an organization would likely prioritize activating alert features based on follow-up preventative actions that the alert is meant to support, such as the protocol screening implemented by the Cleveland Clinic in Table 1.

### Medication management

Avoidable complications in the hospital are frequently related to medication management. The disruption that occurs between a person’s home medication regimen and the regimen they receive while hospitalized can lead to a high rate of medication errors that can cause complications. This is often viewed in the context of MODS - Missed, Omitted, Delayed, and Substituted medications (Parkinson’s Foundation Hospital Care Initiative, 2022). Medications are frequently missed (unintentionally) or delayed, often because of the typical shift in medication timing from the home regimen to the more rigid, default, standard hospital medication distribution schedules (Shin and Habermann, 2016; Mucksavage and Kim, 2020). Medication omission can occur if a specific PD medication is not available in the hospital’s formulary (Derry et al., 2010), or when a patient is not directly admitted for Parkinson’s disease and appropriate medication management is not identified as a primary focus (Shin and Habermann, 2016). Inappropriate omissions have been shown to increase the risk of mortality (Lertxundi et al., 2017). The other frequent result of medications being unavailable on the formulary is inappropriate substitution (Mucksavage and Kim, 2020), which also can be detrimental to PD patients. The second category of complications occurs when potentially harmful medications are administered. In particular, we see complications due to the use of commonly used antipsychotic, antiemetic, antidepressant, analgesic, and anesthetic medications that may interact with Parkinson’s medications and make Parkinson’s symptoms worse (By the 2019 American Geriatrics Society Beers Criteria® Update Expert Panel, 2019), Finally, patients’ swallowing function can be compromised when their Parkinson’s medications are suddenly and inappropriately stopped, putting them at risk of aspiration pneumonia – the leading cause of mortality among people with PD.

A major contribution of this work is the emphasis of the EMDS to advocate for an EHR-based solution to address the urgent need of ensuring that PD medications are given at the same correct frequency and timing as they were taking as an outpatient. We note that three of four example solutions provided by organizations all faced the same outpatient-to-inpatient medication timing limitation (customized timing of PD medication) (Table 1). Developing a more integrated EHR-supported solution became a priority for the EMDS. After extensive discussion, the primary recommendation of enhancing outpatient medication timing to align with inpatient medication timing would require a core change in how the Epic EHR represents outpatient medication frequencies and timing. Such a change is challenging to undertake, requiring significant research and development costs to the vendor. We note that, in addition to the collaborative advocacy of the EMDS, the vendor, after additional support from other specialty steering boards, not limited to neurology, committed to undertake such a change. Preliminary work supports a promising development roadmap.

Avoiding inappropriate medications (neuroleptics) from being ordered for hospitalized PWP remains a gap in the current roadmap. Epic supports standard clinical decision support tools, which can alert the ordering provider of a drug-drug interaction or a drug-disease interactions. Overall, drug–drug interactions are more often enabled by organizations than drug-disease alerts. Organizations need careful governance and review of clinical decision support tools to minimize potential alert fatigue. In the experience of the EMDS members, few default PD-condition alerts existed at all before undertaking quality improvement efforts. In addition, the alerts are based on lists of drug-disease interactions purchased from third party medication vendors. Engagement these vendors is out of scope for the EMDS project and requires additional collaboration between specialty societies and these vendors. Further review suggested that there is not yet a clear standard at a national level to classify the nature of neuroleptic to parkinsonism interactions, and work to establish such a standard was also beyond the scope of this EMDS collaboration. Subject matter experts in the EMDS continue to pursue this work outside of this collaboration.

### PD care plans

As disease diagnosis and treatment paradigms have advanced, so has the complexity of patient management. Translating this complexity into the training of physicians, nurses, and other practitioners can be challenging, with less common neurologic disorders often affected more (Jozefowicz, 1994; Flanagan et al., 2007; Ridsdale et al., 2007; Zinchuk et al., 2010; Willis et al., 2011; Dorsey et al., 2013). With a 1–2% prevalence, the intricacies of managing Parkinson’s disease can be overshadowed by other more common disorders. This has left a knowledge gap in the care workforce (Guttman et al., 2004; Shih et al., 2013). In ongoing work by the EMDS, sample content emphasizing nursing care plans, tools to monitor ancillary services (physical therapy, occupational therapy, speech and language pathology), discharge planning, and discharge instructions is being reviewed and adapted as future standardized content for the Epic Foundation System.

### Advantages

Other advantages to the approach of developing and standardizing tools at the EHR vendor level go beyond automatic deployment or availability of new features to organizational customers. Most EHR vendors now support tools for organizations to suggest new features, monitor adoption of new features, and allow benchmarking of adoption across organizations. Epic publishes a Neurology Success Guide highlighting content specific to the neurology specialty and including links to build and implementation guides. These resources can also be incorporated into an implementation arm of the Parkinson Foundation efforts to improve safety measures for hospitalized PWP, particularly within their Global Care Network.

The development of standardized toolkits for reducing harm in hospitalized PWP has additional benefits in enforcing standardized data collection. For example, the EMDS and Neurology Specialty Steering Board could maintain a diagnosis grouper in Epic’s Foundation System that defines the ICD-10 codes and/or SNOMED terminologies in a standardized definition that all organizations can use. This avoids fragmentation of the cohort of patients that are being addressed by these tools. Across all organizations who use Epic, discrete data fields supporting this toolkit become automatically available to collect information about ancillary services monitored, the activation of a nursing care plan, the benchmarked frequency of inpatient medications ordered with custom timing. These data fields automatically support cross-organizational aggregation of quality metrics.

### Challenges

The lack of awareness and appreciation of the potential positive impact of the available EHR tools in the quality and safety of hospitalized PD patients is the biggest challenge to implementing the currently available and forthcoming solutions. Scientific consensus is needed as a basis to implement solutions; advocacy partnerships, such as with the Parkinson’s Foundation, are essential to aid in prioritization to decide what to implement. As significant bureaucratic obstacles to enabling, configuring, and implementing EHR tools exist, a concerted effort among the movement disorder and neurologist community is required to overcome them. Local organization champions are critical to not only enable build from Epic’s Foundation System in the local EHR, but are also key in adapting the out-of-the-box solutions to fit local organization policies and workflows. Some features require local organizations to develop protocols, policies, and/or procedures to implement changes in workflows or practices to increase the effectiveness of the feature. We hope that the technical development of these features by the EHR vendor can at least reduce some barriers faced for adoption. As a future step, alignment with quality standards or alternative value-based payment models at a national level, could also help incentivize adoption at scale. The current Epic Neurology Specialty Steering Board continues to discuss alignment of future Epic roadmap development with the American Academy of Neurology Quality committees.

Some desired interventions were unable to fit within an EHR vendor roadmap for improvement. There was a limitation in EHR core functionality in aligning medication timing and frequency from outpatient to inpatient settings. Here, we illustrate the importance of collaborative advocacy to help prioritize vendor resources to improve such functionality. Until the development solution becomes available, organizations will continue to struggle with managing PWP medications across the continuum. They will need to design local solutions similar to the three example solutions in Table 1. Another challenge encountered is incorrect or missing drug-disease alerts from third party medication vendors’ content. Specialty societies such as the Parkinson Foundation or American Academy of Neurology should advocate to the medication vendors updates needed to their content. Until the content gaps are addressed, organizations will need to create local clinical decision support solutions.

A major limitation of this work is its focus on a single major EHR vendor which took advantage of the vendor-supported specialty steering board of neurology peers. We hope that first, the toolkit and roadmap described here can incentivize and serve as a model for development and/or adoption by other EHR vendors. Eventually, vendor neutral solutions and resources, such as HL7 standards may adopt and promote some of these tools as requirements for EHR certification. For example, the Agency for Healthcare Research and Quality (AHRQ) supports a framework to represent clinical decision support (CDS) artifacts as a vendor-neutral archive^1^ with CDS artifacts defined as actionable medical knowledge distilled from various evidence sources (e.g., clinical practice guidelines, peer-reviewed articles, local best practices, and clinical quality measures) and translated into computable and interoperable decision support. Tools developed here could be translated into a vendor-neutral “artifact” and stored on this repository for any EHR, organization, or third party vendor to implement in a standard way in the future.

This work illustrates the possible solutions but does not report on the effectiveness of any of the tools to affect outcomes, that is, to actually reduce risk to PWP. We anticipate that work to reduce risks to PWP can only benefit from the broader availability of tools that are deployed as available throughout a large community of organizations that use EHRs that have those standardized tools. Such work holds potential to facilitate collaborative outcomes research by facilitating aggregate interventions based on these tools.

## Conclusion

Preventable harm in the hospital for people living with Parkinson’s disease is often the result of a fragmented healthcare ecosystem in which inpatient care teams may have insufficient PD education and lack recognized care standards and protocols to guide PD treatment. Eliminating these avoidable errors and improving patient safety will require innovation, clinical leadership and commitment, and partnership across healthcare delivery.

With this work, we demonstrated options in use by current organizations, a standardized toolkit of features currently available and forthcoming in the Epic EHR system for improving the safety and quality of care for people with PD in the hospital resulting from the collaboration of an advocacy group with an EHR vendor, with input from subject matter experts across different healthcare systems. We invite healthcare system leaders and other vendors to explore these available tools as a starting point to improve inpatient care for patients with Parkinson’s, and to consider the authors of this paper to be a resource for initiating these interventions and tailoring them to each unique care environment.

## Data availability statement

The original contributions presented in the study are included in the article/supplementary material, further inquiries can be directed to the corresponding authors.

## Author contributions

AW: Writing – original draft, Writing – review & editing, Conceptualization, Methodology, Investigation. BW: Writing – original draft, Writing – review & editing, Conceptualization, Investigation. AB: Writing – original draft, Writing – review & editing, Conceptualization. EB: Writing – original draft, Writing – review & editing. KA: Writing – original draft, Writing – review & editing, Conceptualization. IR: Writing – original draft, Writing – review & editing. KM: Resources, Writing – original draft, Writing – review & editing. HA: Conceptualization, Writing – original draft, Writing – review & editing.

## Footnotes

^1^ https://cds.ahrq.gov/cdsconnect/repository
